# The cytotoxic conjugate of highly internalizing tetravalent antibody for targeting FGFR1-overproducing cancer cells

**DOI:** 10.1186/s10020-021-00306-2

**Published:** 2021-05-07

**Authors:** Marta Poźniak, Natalia Porębska, Mateusz Adam Krzyścik, Aleksandra Sokołowska-Wędzina, Kamil Jastrzębski, Martyna Sochacka, Jakub Szymczyk, Małgorzata Zakrzewska, Jacek Otlewski, Łukasz Opaliński

**Affiliations:** 1 Faculty of Biotechnology, Department of Protein Engineering, University of Wroclaw, Joliot‐Curie 14a, 50‐383 Wroclaw, Poland; 2 Laboratory of Cell Biology, International Institute of Molecular and Cell Biology, Warsaw, Poland

**Keywords:** FGFR, ADC, Conjugate, Cytotoxic, Cancer

## Abstract

**Background:**

Antibody drug conjugates (ADCs) represent one of the most promising approaches in the current immuno-oncology research. The precise delivery of cytotoxic drugs to the cancer cells using ADCs specific for tumor-associated antigens enables sparing the healthy cells and thereby reduces unwanted side effects. Overexpression of fibroblast growth factor receptor 1 (FGFR1) has been demonstrated in numerous tumors and thereby constitutes a convenient molecular target for selective cancer treatment. We have recently engineered tetravalent anti-FGFR1 antibody, T-Fc, and have demonstrated that it displays extremely efficient internalization into FGFR1 producing cells, a feature highly desirable in the ADC approach. We have revealed that T-Fc mediates clustering of FGFR1, largely enhancing the uptake of FGFR1-T-Fc complexes by induction of clathrin-independent endocytic routes. The aim of this study was to obtain highly internalizing cytotoxic conjugate of the T-Fc for specific delivery of drugs into FGFR1-positive cancer cells.

**Methods:**

Conjugation of the T-Fc to a cytotoxic payload, vcMMAE, was carried out via maleimide chemistry, yielding the T-Fc-vcMMAE. The specific binding of the T-Fc-vcMMAE conjugate to FGFR1 was confirmed in vitro with BLI technique. Confocal microscopy and flow cytometry were applied to determine FGFR1-dependence of the T-Fc-vcMMAE internalization. Western blot analyses of FGFR1-dependent signaling were conducted to assess the impact of the T-Fc-vcMMAE on FGFR1 activation and initiation of downstream signaling cascades. Finally, using FGFR1-negative and FGFR1-possitive cell lines, the cytotoxic potential of the T-Fc-vcMMAE was evaluated.

**Results:**

We have performed the efficient conjugation of the tetravalent engineered antibody with a cytotoxic drug and generated FGFR1-specific ADC molecule, T-Fc-vcMMAE. We have demonstrated that T-Fc-vcMMAE conjugate exhibits high selectivity and affinity for FGFR1, similarly to T-Fc. Furthermore, we have shown that T-Fc constitutes an effective drug delivery vehicle as T-Fc-vcMMAE was efficiently and selectively internalized by FGFR1-producing cells leading to their death. Interestingly, we show that the efficiency of the uptake of T-Fc-vcMMAE corresponds well with the cytotoxicity of the conjugate, but doesn’t correlate with the FGFR1expression level.

**Conclusion:**

Our results show that T-Fc-vcMMAE fulfills the key criteria for the successful cytotoxic drug carrier in a targeted approach against FGFR1-positive cancer cells. Furthermore, our data implicate that not solely expression level of the receptor, but rather its cellular trafficking should be taken into account for selection of suitable molecular targets and cancer models for successful ADC approach.

## Background

The drawback of conventional anticancer drugs that are currently in the clinical use is their off-target toxicity, as typically these compounds affect proliferating cells, including healthy ones. A major advancement in the cancer treatment is development of the concept of targeted therapies, where therapeutic modalities are based on the specific molecular characteristics of a patient's tumor (Hallinan et al. 2016; Chari 2008). The ultimate aim of the targeted anti-cancer therapies is the precise delivery of cytotoxic drugs into cancer cells, sparing the healthy ones and thus limiting unwanted side effects (Lee et al. 2018; Yan et al. 2011).

Currently, one of the most promising targeted therapeutics are antibody–drug conjugates (ADCs). Typically, ADC consists of a monoclonal antibody (mAb), specific for an antigen expressed on tumor cells, that is linked to a highly cytotoxic drug by a specific linker, which controls release of the drug inside targeted cells, but not in the bloodstream. The mAb ensures the specificity of ADC and facilitates its efficient intracellular delivery. Upon binding to the targeted antigen, ADC is taken up to the cell interior by receptor-mediated endocytosis and delivered to lysosomes via sophisticated intracellular vesicular transport system (Fauvel and Yasri 2014; Kim and Kim 2015; Sobhani et al. 2018; Chau et al. 2019; Sokolowska-Wedzina et al. 2017). In lysosomes the specific linker is cleaved and the proteinaceous part of ADC is proteolytically degraded. The cytotoxic drug moiety released from decomposed ADC crosses endomembranes and reaches the intracellular targets (Coats et al. 2019; Kim et al. 2019).

Fibroblast growth factor receptors (FGFRs) that constitute a group of four receptor tyrosine kinases (RTKs) (FGFR1-FGFR4) emerged recently as attractive molecular targets for selective cancer treatment (Porębska et al. 2018; Dieci et al. 2013; Szlachcic et al. 2016). Aberrations in the FGFRs-FGFs system are frequently associated with many developmental syndromes and progression of different types of cancer (Hallinan et al. 2016; Carter et al. 2015; Fearon et al. 2013; Haq et al. 2018). The increased level of FGFR1 was found in numerous tumors, including breast, lung, head, and neck cancers, and is predictor of poor outcome in patients (Elsheikh et al. 2007; Jang et al. 2012; Murphy et al. 2010; Peifer et al. 2012; Weiss et al. 2010; Tomlinson et al. 2009).

FGFR1 is subjected to the constitutive low-rate internalization from the plasma membrane. Binding of FGFs leads to FGFR1 dimerization that largely accelerates cellular uptake of the receptor predominantly through clathrin-mediated endocytosis (CME) (Auciello et al. 2013; Haugsten et al. 2005; Opalinski et al. 2017). We have recently constructed an engineered tetravalent antibody, T-Fc, capable of clustering FGFR1 into high molecular weight complexes. FGFR1 oligomerization induced by the T-Fc largely improved the internalization of the receptor by stimulating not only CME, but also clathrin-independent endocytosis (CIE) that requires activity of dynamin-2 (Pozniak et al. 2020). It was demonstrated that cancer cells may tune the activity of distinct endocytic pathways depending on environmental conditions (Roepstorff et al. 2008; Hinze and Boucrot 2018). For example, constantly dividing cancer cells may increase the lifetime of activated RTKs on the cell surface and promote oncogenic signaling by downregulating CME (Fielding and Royle 2013). Since the T-Fc employs multiple endocytic pathways, T-Fc-FGFR1 complexes are less prone to alterations in protein trafficking in cancer cells, suggesting that the T-Fc could constitute highly attractive drug carrier for ADC approach.

Here, we have tested the potential of the T-Fc as a drug delivery platform for the treatment of FGFR1-positive cancers. We have constructed the cytotoxic conjugate, T-Fc-vcMAME, by incorporating valine-cytruline linker-bearing monomethyl auristatin E (vcMMAE) to the cysteines of the Fc fragment of T-Fc. We have shown that the T-Fc conjugated to vcMMAE retains high specificity and affinity for FGFR1 and is efficiently internalized into FGFR1-expressing cells via receptor-mediated endocytosis. Importantly, we have positively evaluated T-Fc as an effective drug delivery vehicle as the T-Fc-vcMMAE displayed potent and selective cytotoxicity against FGFR1-positive cancer cells.

## Methods

### Antibodies and reagents

The primary antibodies directed against FGFR1 (#9740), phospho-FGFR (pFGFR; #3476), ERK1/2 (#9102) and phospho-ERK1/2 (pERK1/2; #9101) were from Cell Signaling (Danvers, MA, USA). Anti-tubulin primary antibody (#T6557) was from Sigma-Aldrich (St Louis, MO, USA) and anti-human IgG (Fc) antibody coupled to HRP (#4-10-20) was from KPL (Gaithersburg, MA, USA). Secondary antibodies coupled to HRP were from Jackson Immuno-Research Laboratories (Cambridge, UK).

### Recombinant proteins

Extracellular domain of FGFRs fused to the Fc fragment of human IgG1: FGFR1 IIIc (FGFR1-Fc) was produced as described previously by our group (Sokolowska-Wedzina et al. 2014). T-Fc was expressed and purified according to (Pozniak et al. 2020).

### Cells

Human osteosarcoma cell line (U2OS) was obtained from American Type Culture Collection (ATCC). U2OS-R1 cells were obtained using U2OS cell line that was transfected with pcDNA3 vector containing sequence encoding full length FGFR1 using Fugene reagent (Roche, Indianapolis, IN, USA). The cells were re-plated 24 h after transfection and cultured in selection media (growth media with 1 mg/mL geneticin) until colony formation was observed. Colonies were transferred using cloning discs (Sigma-Aldrich) to 6-well plates, and then to T-75 cm^2^ flask for continued culture. Expression of FGFR1 was confirmed by western blotting using antibody against FGFR1. Cells were cultured in Dulbecco’s Modified Eagle’s Medium (DMEM) (Biowest, Nuaille, France) supplemented with 10% fetal bovine serum (FBS) (Thermo Fisher Scientific, Waltham, MA, USA), antibiotics mix (100 U/mL penicillin and 100 μg/mL streptomycin) (Thermo Fisher Scientific), for U2OS-R1 additionally supplemented with 1 mg/mL geneticin (Thermo Fisher Scientific). Murine embryonic fibroblasts (NIH3T3) were from ATCC and were cultured in DMEM (Biowest, Nuaille, France) supplemented with 2% bovine serum (BS) (Thermo Fisher Scientific) and antibiotics mix (100 U/mL penicillin and 100 μg/mL streptomycin). Human lung cancer cell line NCI-H520, breast cancer cell line NCI-H1581 and breast cancer cell line T47D were obtained from ATCC. Non-small lung cancer cell line HCC15, breast cancer cell line JIMT-1 and lung adenocarcinoma cell line COLO-699 were supplied by the Leibniz Institute DSMZ-German Collection of Microorganisms and Cell Cultures (DSMZ). NCI-H520 cells were cultured in RPMI 1640 Medium (ATCC) supplemented with 10% FBS and antibiotics mix (100 U/mL penicillin and 100 μg/mL streptomycin). NCI-H1581, HCC-15 and COLO-699 cell lines were cultured in RPMI 1640 Medium (Biowest) supplemented with 10% FBS and antibiotics mix (100 U/mL penicillin and 100 μg/mL streptomycin). JIMT-1 cell line was cultured in DMEM supplemented with 10% FBS and antibiotics mix (100 U/mL penicillin and 100 μg/mL streptomycin), and T47D cell line was cultured in RPMI 1640 Medium (ATCC) supplemented with 10% FBS, 0.2 U/mL insulin and antibiotics mix (100 U/mL penicillin and 100 μg/mL streptomycin). All cell lines were grown in 5% CO_2_ atmosphere at 37 °C. Cells were seeded onto tissue culture plates one day prior the start of the experiments.

### Conjugation of vcMMAE to the T-Fc

Conjugation of the T-Fc with cytotoxic payload was performed in reaction buffer (50 mM NaCl, 18 mM NaH2PO4, 33 mM Na2HPO4 pH, 5% glycerol, 1 mM EDTA, 1 M urea, pH 6.8). Disulfide bonds within the hinge region of T-Fc were reduced by adding tris(2-carboxyethyl) phosphine (TCEP) pH 7.0 in ten-fold molar excess over protein and incubating for 1 h, RT. Then reduced and 10 times diluted by reaction buffer T-Fc was added to maleimidocaproyl-Val-Cit-PABC-monomethyl auristatin E (vcMMAE) (MedChem Express, Monmouth Junction) in 15-fold molar excess over protein -SH group and incubated for 2 h at 16 °C. The T-Fc-vcMMAE was purified by ion exchange chromatography using HiTrap CM Sepharose FF column (GE Healthcare, Chicago, IL, USA). The resin was washed with washing buffer (10 mM MES pH 6.2) to remove unconjugated vcMAME and then the T-Fc-vcMMAE was eluted with the elution buffer (10 mM sodium citrate, 494 mM NaCl, 6 mM KCl, 5% glycerol, 0.1% PEG 3350, pH 5.6). The purity and the identity of conjugate were confirmed by SDS-PAGE and western blotting. The Drug to Antibody Ratio (DAR) was determined spectrophotometrically according to (Chen 2013). The UV–Vis spectra were recorded in the 240–320 nm range in the elution buffer and the following extinction coefficients ε_mmae_^248^ = 15,900 L/mol cm^−1^, ε_mmae_^280^ = 1500 L/mol cm^−1^, ε_T-Fc_^248^ = 24,627 L/mol · cm^−1^ and ε_T-Fc_^280^ = 36,330 L/mol cm^−1^ were used.

### BLI measurements

Binding of the T-Fc and the T-Fc-vcMMAE to FGFR1 was measured using bio-layer interferometry (BLI) with ForteBio Octet K2 (Pall ForteBio, San Jose, CA, USA). The extracellular region of FGFR1 fused to the Fc-fragment of human IgG1 (FGFR1-Fc) (10 μg/mL) was chemically immobilized on Amine Reactive Second-Generation (AR2G) biosensors (Pall ForteBio, San Jose, CA, USA). The measurements were conducted at 25ºC for the T-Fc (10 μg/mL) and the T-Fc-vcMMAE (10 μg/mL) in PBS supplemented with 0.2% BSA and 0.05% Triton X-100. Both association and dissociation phases were monitored by 300 s.

### SPR measurements

SPR experiments were performed on the Biacore 3000 instrument (GE Healthcare) at 25 °C in PBS with 0.05% Tween 20, 0.02% NaN_3_, pH 7.2. To determine the kinetics of T-Fc-MMAE binding to FGFR1, various concentrations of the conjugate (0.625–10 nM) were injected on the CM4 sensor chip with immobilized the extracellular region of FGFR1 fused to the Fc fragment of human IgG1 at 1000 RU for 120 s at 30 μL·min^−1^ flow rate. The dissociation was monitored for next 180 s, and 10 mM glycine, pH 1.5 was applied for sensor regeneration. Kinetic constants (*k*_on_, *k*_off_, and *K*_D_) were calculated using BIAevaluation 4.1 software (GE Healthcare) using 1: 1 Langmuir binding model with drifting baseline.

To examine the interaction of antibody alone and its conjugate with murine FGFR1, each compound (40 nM) was injected on the CM4 sensor chip with immobilized murine recombinant FGFR1 (10,135-FR, R&D Systems) at 535 RU for 120 s at 30 μL·min^−1^ flow rate. The dissociation was monitored for next 180 s, and 10 mm glycine, pH 1.5 was applied for sensor regeneration. The binding curves were analyzed using BIAevaluation 4.1 software (GE Healthcare).

### Activation of FGFR1 and receptor-downstream signaling cascades

To analyze the impact of T-Fc and T-Fc-vcMAME on FGFR1 activation and initiation of receptor-downstream signaling cascades, serum starved NIH3T3 cells were incubated for 15 min at 37ºC with T-Fc (1, 2, 5 μg/mL), T-Fc-vcMMAE (1, 2, 5 μg/mL) or FGF1 (100 ng/mL) in the presence of heparin (10 U/mL). Cells were lysed in Laemmli buffer and subjected to SDS-PAGE and western blotting.

### Fluorescence microscopy

For the analysis of T-Fc and T-Fc-vcMMAE cellular uptake, early endosomes in U2OS-R1 cells were labeled with Rab5a-RFP (CellLight Early Endosomes-RFP, Thermo Fisher Scientific) and cells were incubated with proteins (both at 100 nM) at 37 °C for 15 min in the presence of 5% CO_2_. The internalization was stopped by cooling down the cells on ice. Next, cells were fixed with 4% paraformaldehyde and permeabilized with 0.1% Triton in PBS. Zenon AF488 (Thermo Fisher Scientific) was used for labeling of the T-Fc and the T-Fc-vcMMAE, and NucBlue Live (Thermo Fisher Scientific) was used for fluorescent labeling of nuclei. For analysis of the T-Fc uptake by different cell lines, cells were incubated with T-Fc (100 nM) for 30 min at 37 °C and processed as described above. Wide-field fluorescence microscopy was carried out using Zeiss Axio Observer Z1 fluorescence microscope (Zeiss). Images were taken using LD-Plan-Neofluar 40 × /0.6 Korr M27 objective and Axiocam 503 camera. Zenon AF-488 signal was visualized with a 450/490 nm bandpass excitation filter and a 500/550 nm bandpass emission filter. CellLight Early Endosomes-RFP signal was visualized with a 540/552 nm bandpass excitation filter and a 575/640 nm bandpass emission filter. NucBlue Live signal was visualized with 335/383 nm bandpass excitation filter and 420/470 nm emission filter. Images were processed with Zeiss ZEN 2.3 software (Zeiss, Oberkochen, Germany) and Adobe Photoshop (Adobe, San Jose, CA, USA).

The quantitative internalization of T-Fc or T-Fc-vcMMAE was analyzed with immunofluorescence protocol and confocal microscopy. U20S-R1 cells were seeded at density 8 × 10^3^ per well on µClear 96-well plates from Greiner Bio-One (Kremsmunster, Austria) (#655,096). After 24 h, U2OS-R1 cells were incubated either with T-Fc or T-Fc-MMAE (both at 100 nM) for indicated time points at 37 °C. Following stimulation, cells were transferred to ice and washed with ice-cold PBS. To measure binding of T-Fc or T-Fc-vcMMAE to cell surface, cells were stimulated with precooled T-Fc or T-Fc-vcMMAE for 5 min on ice (0 min time point on the graphs). Then cells were processed as described previously with some modifications. At least twenty 16-bit images with resolution 2048 × 2048 pixels and were acquired per experimental condition.

### Flow cytometry

U2OS-R1 cells were seeded onto 6-well plates (150 000 cells per well) in full medium and left to attach overnight. Next day the medium was removed, cells were washed with PBS buffer and starved with serum-free medium for 4 h. Next, plates were cooled on ice, and T-Fc (500 ng/mL) or the T-Fc-vcMMAE (500 ng/mL) labeled with DyLight 550 (Thermo Fisher Scientific) were added to the cells in serum-free medium supplemented with 1% BSA to allow for FGFR1 binding without internalization (endocytosis is blocked due to the low-temperature-dependent decrease in membrane dynamics). After 40 min of incubation on ice, the cells were moved to 37 °C for 15 min to allow for internalization. Then, the medium was removed and the cells were washed with serum-free medium supplemented with 0.2% BSA pH 3.5 (three times, 5 min) and then with PBS buffer (three times, 1 min) to remove non-internalized, cell-surface bound proteins. Cells were subsequently detached with 10 mM EDTA in PBS buffer, pH 8.0, harvested by centrifugation and resuspended in PBS supplemented with 1% BSA. Cells were analyzed using a NovoCyte 2060R Flow Cytometer and NovoExpress software (ACEA Biosciences, San Diego, CA).

### Cytotoxicity assays

The cytotoxicity of the T-Fc-vcMMAE was evaluated on FGFR1-negative cell lines (U2OS, HCC-15, T47D) and FGFR1-positive cell lines (U2OS-R1, NCI-H520, JIMT-1, COLO-699, NCI-H1581). Cells were plated (5000 cells per well) in 96-well plates and incubated for 24 h at 37 °C in the presence of 5% CO_2_. Serial dilutions of T-Fc (control), T-Fc-vcMMAE (from 0.001 to 1000 nM) and equimolar concentration of free MMAE (from 0.004 to 4000 nM) were incubated with the cells for 96 h. Cell viability was measured using PrestoBlue™ Cell Viability Reagent (Thermo Fisher Scientific), according to the manufacturer's protocol. Fluorescence emission at 590 nm (upon excitation at 560 nm), reflecting the viability of the cells, was measured using Infinite M1000 PRO plate reader (Tecan, Männedorf, Switzerland). Every experiment was conducted in triplicates. EC_50_ values were calculated based on the Hill equation using Origin 7 software (Northampton, MA). Statistical analyses were done using t-test by comparing viability of untreated cells with viability of cells upon treatment with proteins or conjugates.

## Results

### Engineering of the cytotoxic conjugate T-Fc-vcMMAE

**Fig. 1:**
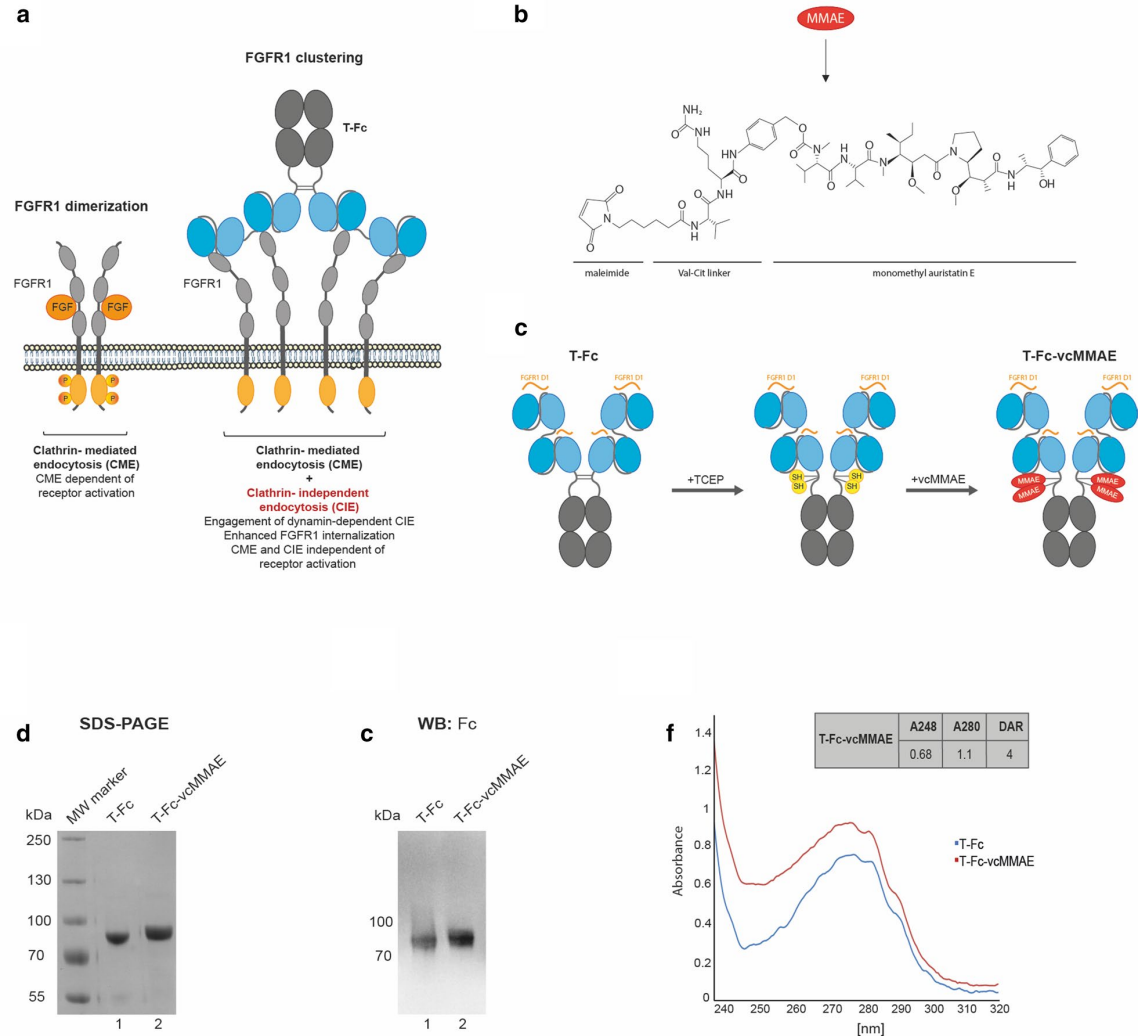
Conjugation of tetravalent engineered antibody (T-Fc) with a cytotoxic payload. **a** Hypothetical model of the effect of FGFR1 clustering on the receptor endocytosis. FGFR1 dimerization via FGF1 binding induces receptor activation and clathrin-mediated endocytosis. Clustering of FGFR1 into large structures on the plasma membrane with tetravalent T-Fc largely improves the cellular uptake of FGFR1-antibody complexes. Furthermore, FGFR1 clustering changes the mechanism of the receptor endocytosis by engaging dynamin-2-dependent CIE pathways. **b** The chemical structure of monomethyl auristatin E bearing the valine-citrulline linker (vcMMAE). **c** The schematic representation of the conjugation of T-Fc with the cytotoxic compound MMAE. The Fc region of IgG (CH2 and CH3 domains) is labeled in gray, and anti-FGFR1 scFv proteins (VH and VL fusions) are marked in blue. Antibody regions recognizing epitopes within FGFR1 are marked in orange. Thiol groups of reduced cysteines are marked in yellow and attached cytotoxic payloads are marked in red. **d**, **e** The efficiency of the conjugation and purity of T-Fc-vcMMAE were analyzed with SDS/PAGE (**d**) and western blotting (**e**) with antibodies recognizing the Fc fragment. **f** The spectroscopic analysis of DAR parameter for T-Fc-vcMMAE. DAR was calculated through the absorbance measurement for T-Fc and T-Fc-vcMMAE at 248 nm and 280 nm wavelengths according to (Chen 2013).

We have recently reported the development of a tetravalent anti-FGFR1 engineered antibody, T-Fc. T-Fc displayed extremely high affinity and selectivity towards FGFR1. Importantly, we have shown that T-Fc-mediated FGFR1 clustering on the cell surface boosts the endocytosis of T-Fc/FGFR1 complexes. Using siRNA-mediated knock-down of distinct endocytic proteins in conjunction with quantitative confocal microscopy we have found that T-Fc/FGFR1 clusters are internalized by simultaneous engagement of multiple endocytic pathways, including clathrin-mediated endocytosis and clathrin-independent, dynamin-2-dependent endocytic routes (Fig. 1a) (Pozniak et al. 2020). Thus, our data suggested that the T-Fc may constitute a highly efficient drug delivery vehicle for selective treatment of FGFR1-dependent cancers using ADC approach.

To verify our assumption, we conjugated T-Fc in a site-specific manner to a potent antimitotic agent, monomethyl auristatin E, bearing the valine-citrulline linker (vcMMAE) (Fig. 1b, c). MMAE blocks cell division by inhibiting tubulin polymerization, while valine-citrulline linker ensures an efficient release of protein-bound MMAE by lysosomal proteolysis with cathepsin B inside the target cell (Caculitan et al. 2017; Chen et al. 2017). Essentially, by covalent coupling of vcMMAE to the thiol groups of TCEP-reduced cysteines present within the Fc fragment of T-Fc we obtained T-Fc-vcMMAE (Fig. 1c). The efficiency of the conjugation process was monitored with SDS-PAGE. The attachment of vcMMAE to T-Fc caused decreased in-gel mobility and confirmed high efficiency of the conjugation reaction, as virtually no unconjugated T-Fc was detected (Fig. 1d, lane 2). The identity of obtained T-Fc-vcMMAE was further confirmed with western blotting using anti-Fc antibodies (Fig. 1e).

In principle four cysteine residues within the T-Fc are available for conjugation (Fig. 1c). To gain insights into the molecular architecture of the T-Fc-vcMMAE conjugate we determined drug-to-antibody ratio (DAR) using spectroscopic measurements in the UV-VIS range according to the established protocol (Chen 2013). DAR in T-Fc-vcMMAE conjugate was equal to 4, implicating that in most cases four cytotoxic payload molecules are attached to a single T-Fc molecule (Fig. 1f).

All these data demonstrate the successful development of T-Fc-vcMMAE, the conjugate of a highly internalizing anti-FGFR1 antibody T-Fc, with a potent cytotoxic drug, MMAE.

### FGFR1 binding and receptor-mediated internalization of the T-Fc-vcMMAE conjugate

**Fig. 2:**
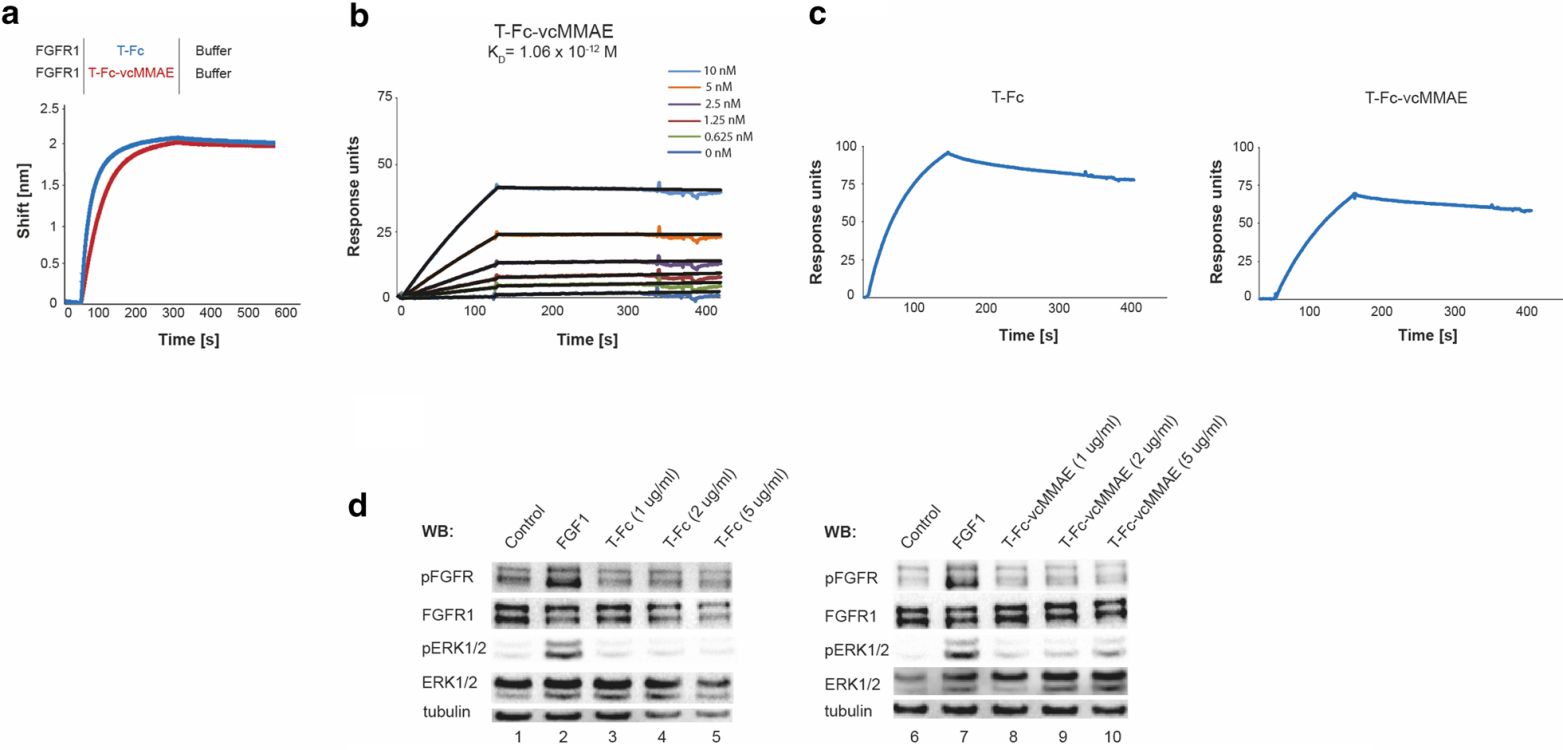
Interaction of T-Fc-vcMMAE with FGFR1. **a** Evaluation of T-Fc and T-Fc-vcMMAE interaction with FGFR1 by BLI. The extracellular region of FGFR1 was immobilized on BLI sensors and incubated either with T-Fc or T-Fc-vcMMAE. The association and dissociation profiles were measured. **b** SPR-determined kinetic parameters of the interaction between T-Fc-vcMMAE and FGFR1. The extracellular region of FGFR1 was immobilized on SPR sensors and incubated with various concentrations of T-Fc-vcMMAE. K_D_ value is presented. **c** SPR results of the interaction between T-Fc and T-Fc-vcMMAE, and murine FGFR1, respectively. The murine recombinant FGFR1 was immobilized on SPR sensors and incubated with T-Fc or T-Fc-vcMMAE. The association and dissociation profiles were measured.** d** T-Fc and T-Fc-vcMMAE are unable to activate FGFR1. Serum-starved NIH3T3 cells were incubated with FGF1 (positive control) or with different concentrations of T-Fc or T-Fc-vcMMAE. Cells were lysed and activation of FGFR1, and receptor-downstream signaling was assessed with western blotting (WB). The level of tubulin served as a loading control

Our previous surface plasmon resonance (SPR) and biolayer interferometry (BLI) analyses revealed that T-Fc binds FGFR1 with picomolar affinity (Pozniak et al. 2020). To study whether incorporation of cytotoxic payload affected T-Fc interaction with FGFR1 we applied BLI method. To this end, FGFR1 was chemically immobilized on biosensors and incubated with T-Fc or T-Fc-vcMMAE. As shown in Fig. 2a the FGFR1 binding curves for the T-Fc and the T-Fc-vcMMAE were almost identical, suggesting that conjugation of vcMMAE has no impact on engineered antibody’s interaction with the receptor. Next, we determined the kinetic parameters of T-Fc-vcMMAE-FGFR1 interaction using SPR. The measured K_D_ value of the conjugate was 1.06 × 10^–12^ and was highly similar to the unconjugated protein (Fig. 2b; Pozniak et al. 2020).

We have recently shown that clustering of the cell surface FGFR1 by T-Fc did not result in FGFR1 activation and initiation of receptor-downstream signaling cascades (Pozniak et al. 2020). Since T-Fc recognizes N-terminal D1 domain of the receptor and not the ligand binding pocket formed by the D2 and D3 domains, it is likely that FGFR1 molecules within T-Fc-induced receptor cluster are in different conformation than in FGF-induced dimer, and thus remain kinase-inactive (Pozniak et al. 2020). Importantly, the lack of FGFR1 activation by T-Fc is a highly desirable feature for drug carrier in the ADC approach, as binding of the therapeutic agent to the surface receptor and drug delivery should not been accompanied by stimulation of cell proliferation. To study the impact of T-Fc-vcMMAE conjugate on FGFR1 activation we employed model murine fibroblasts NIH3T3. First, with SPR we confirmed that T-Fc and T-Fc-vcMMAE bind murine FGFR1 (Fig. 2c). Next, with western blotting we demonstrated that the cytotoxic conjugate (Fig. 2d, lanes 8–10, similarly to T-Fc (Fig. 2d, lanes 3–5), was unable to activate FGFR1 and downstream kinase ERK1/2 at any concentrations tested.

**Fig. 3:**
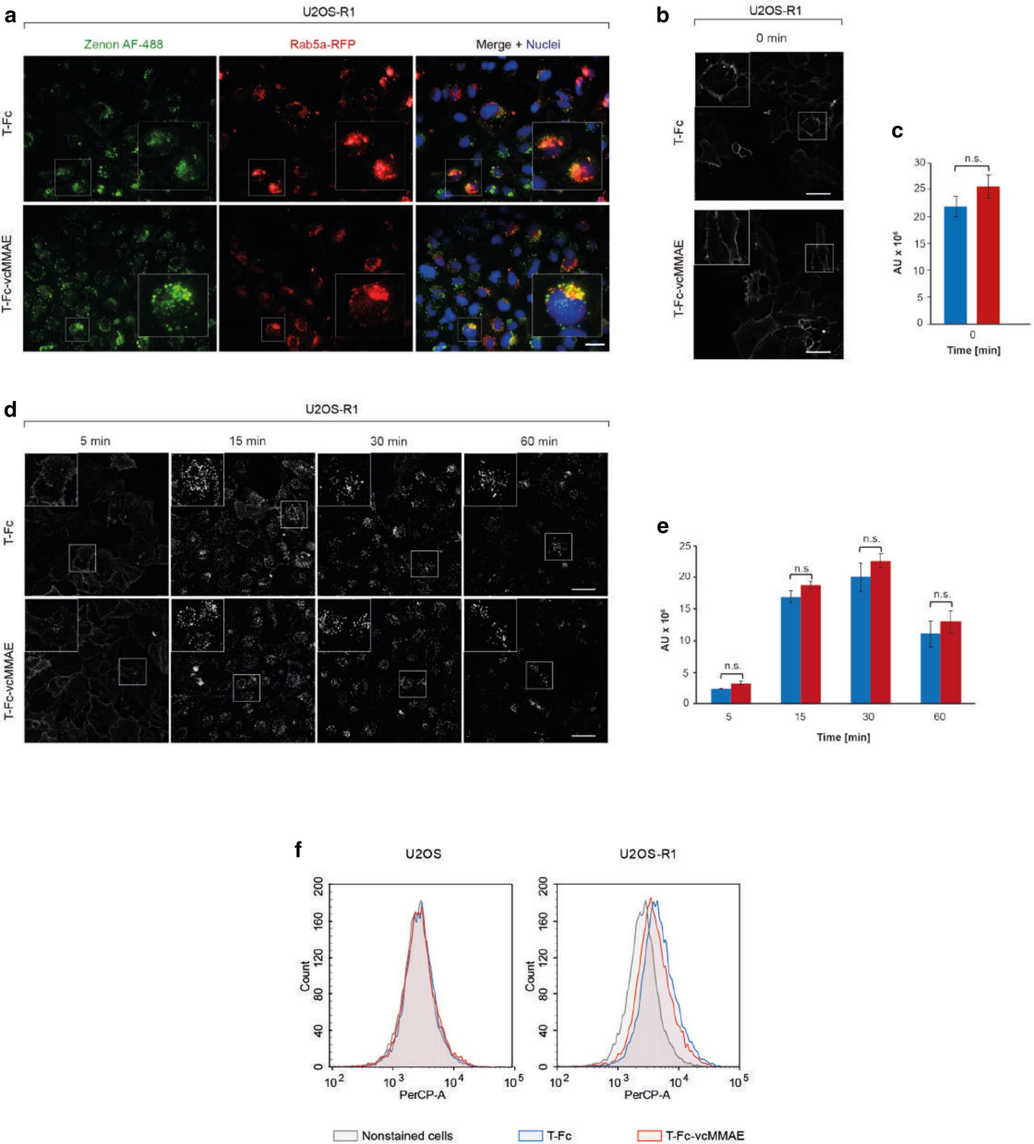
FGFR1-mediated internalization of T-Fc and T-Fc-vcMMAE. **a** FGFR1-dependent endocytosis of T-Fc and T-Fc-vcMMAE. U2OS-R1 cells stably expressing FGFR1 were incubated with T-Fc or T-Fc-vcMMAE for 15 min at 37 °C. Nuclei were stained with NucBlue Live, and early endosomes were labeled by CellLight Early Endosomes-RFP. Cells were fixed, and internalized antibodies were visualized with Zenon AF-488 using wide-field fluorescence microscope. Scale bar represents 20 μm. **b**–**e** Confocal microscopy analysis of the T-Fc and T-Fc-vcMMAE internalization. *B and C*. U2OS-R1 cells were briefly incubated with T-Fc and T-Fc-vcMMAE (t = 0 min) and analyzed with quantitative immunofluorescence microscopy using Zenon-AF-488 to label Fc-bearing recombinant proteins. **d**, **e** T-Fc and T-Fc-vcMMAE were incubated with U2OS-R1 cells for different time periods (5, 15, 30, 60 min) and internalized antibodies were labeled with Zenon AF-488, and analyzed with confocal microscopy. Scale bar represents 50 μm. Quantification of T-Fc and T-Fc-vcMMAE internalization (expressed as integral fluorescence intensity in arbitrary units, AU) was performed using the HARMONY software. Mean values of three independent experiments of integral intensity of Zenon AF-488 signal (**c**) and integral intensity of Zenon AF-488 vesicles (**e**)  ± SEM are shown. *T*-test was used to assess the statistical significance of measured differences in internalization; *p < 0.05, **p < 0.01, ***p < 0.0001, n.s.- not significant. **f** Efficiency and selectivity of T-Fc and T-Fc-vcMMAE internalization studied with flow cytometry. Internalization was analyzed with serum-starved U2OS and U2OS-R1 cells, treated with T-Fc or T-Fc-vcMMAE labeled with DyLight550. After 40 min incubation on ice, cells were transferred to 37 °C for 15 min, and then subsequently analyzed by flow cytometry

The effective receptor-mediated endocytosis is a prerequisite feature of an antibody suitable for ADC (Birrer et al. 2019). Our recent quantitative confocal microscopy analyses in conjunction with knock-down of endocytic pathways revealed that T-Fc-mediated FGFR1 clustering efficiently triggers receptor endocytosis and directs T-Fc/FGFR1 complexes to lysosomes for degradation (Pozniak et al. 2020). Importantly, we have shown that the highly enhanced FGFR1 uptake induced by T-Fc occurs through the simultaneous stimulation of multiple endocytic routes (Pozniak et al. 2020). The highly effective and selective internalization of T-Fc suggested that this engineered antibody may constitute an ideal drug delivery vehicle for FGFR1-overproducing cancer cells. Therefore, we studied the impact of vcMMAE incorporation into T-Fc on the conjugate’s endocytosis using fluorescence microscopy. FGFR1-overproducing U2OS cells (U2OS-R1), initially transfected with CellLight. Early Endosomes-RFP reagent to label early endosome compartments with Rab5a-RFP, were incubated with T-Fc or T-Fc conjugate (T-Fc-vcMMAE) and internalized engineered antibodies were detected using Zenon AF-488. As shown in Fig. 3a, 15 min incubation of cells with T-Fc and T-Fc-vcMMAE resulted in the appearance of highly intense intracellular signal that largely co-localized with Rab5a-RFP. Importantly, the intensities of intracellular T-Fc and T-Fc-vcMMAE signals were virtually identical.

To quantitatively measure T-Fc and T-Fc-vcMMAE endocytosis we employed confocal microscopy. Initially, we have shown that at zero time point both studied proteins accumulated on the cell surface, as expected (Fig. 3b). The intensities of cell surface signals of T-Fc and T-Fc-vcMMAE were highly similar, which is in agreement with SPR and BLI FGFR1 binding studies (Fig. 3c). Next, we quantitatively studied the kinetics and efficiency of T-Fc and T-Fc-vcMMAE internalization. As shown in Fig. 3d, e the uptake of T-Fc and T-Fc-vcMMAE was practically the same. We confirmed microscopy data with flow cytometry, where besides U2OS-R1 cells, we also used U2OS cell line with undetectable FGFR1 expression as the control of uptake specificity. As shown in Fig. 3f no intracellular signal was detected in U2OS cells for both T-Fc and T-Fc-vcMMAE. In contrast, in U2OS-R1 cells fluorescent signal of similar intensity was detected for both T-Fc alone and its conjugate (Fig. 3f).

Above results show that site-specific incorporation of vcMMAE cytotoxic payload into T-Fc has no impact on engineered antibody’s interaction with FGFR1. Furthermore, the T-Fc conjugated to vcMMAE retains high efficiency of FGFR1-mediated internalization.

### The cytotoxic effect of T-Fc-vcMMAE conjugate in FGFR1-possitive cancer cells

**Fig. 4:**
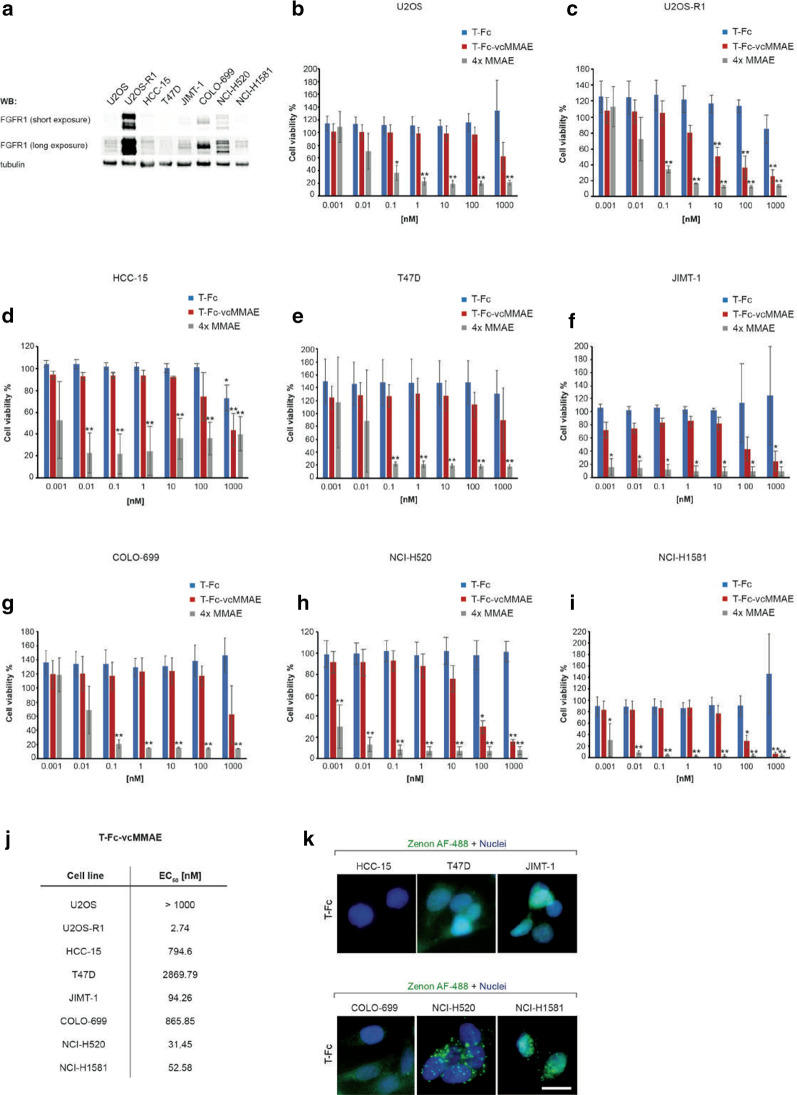
Cytotoxicity of T-Fc-vcMMAE against FGFR1-overproducing cancer cells. **a** FGFR1 expression levels in studied cell lines analyzed by western blotting using anti-FGFR1 antibody. Tubulin level assessed with anti-tubulin antibody served as a loading control. **b**–**i** Cytotoxic potential of T-Fc and T-Fc-vcMMAE. Negative-FGFR1 cells: U2OS (**b**), HCC15 (**d**) and T47D (**e**), and positive-FGFR1 cells: U2OS-R1 (**c**), JIMT-1 (**f**), COLO-699 (**g**), NCI-H520 (**h**) and NCI-H1581 (**i**) were treated with indicated agents at various concentrations for 96 h and their viability was assessed with the Presto Blue assay. Presented results are mean values from three experiments ± SD. Statistical significance: *p < 0.05, **p < 0.01, ***p < 0.0001, not significant differences are not marked. **j** EC_50_ values for T-Fc-vcMMAE for each cell line, respectively. EC50 values were calculated based on the Hill equation using Origin 7 software (Northampton, MA). **k** The T-Fc internalization into studied cancer cell lines, visualized with fluorescence microscopy. Cell line tested in cytotoxic assays were incubated with 15 µg/mL of T-Fc for 30 min at 37 °C. Nuclei were stained with NucBlue Live, cells were fixed, and internalized antibodies were visualized with Zenon AF-488 using wide-field fluorescence microscopy. Scale bar represents 20 μm

To assess the toxicity of T-Fc-vcMMAE, we used a panel of cell lines differing in the level of FGFR1 expression. We applied cells devoid of detectable FGFR1 level: U2OS (osteosarcoma), HCC-15 (lung squamous cell carcinoma) and T47D (invasive ductal carcinoma) as controls, and U2OS-R1 (osteosarcoma cells stably transfected with FGFR1), NCI-H520 (lung squamous cell carcinoma), JIMT-1 (ductal breast carcinoma), COLO-699 (lung adenocarcinoma) and NCI-H1581 (lung large cell carcinoma) cells producing different levels of FGFR1 (Fig. 4a). As shown in Fig. 4b–j T-Fc-vcMMAE did not show significant cytotoxic properties towards control U2OS cell line. For HCC-15 and T47D cells we observed partial cytotoxicity at the highest concentration tested. Since this effect was also observed in the case of T-Fc, it suggests some cytotoxicity of engineered antibody alone at high concentration. Alternatively, there might be some target-independent toxicity of MMAE released from the conjugate. In contrast, T-Fc-vcMMAE exhibited cytotoxic activity against FGFR1-positive cells lines: U2OS-R1, NCI-H520, JIMT-1 and NCI-H1581 in the concentration-dependent manner. The cytotoxic effect of T-Fc-vcMMAE was the most pronounced for U2OS-R1 cells, which exhibit very high FGFR1 level (EC_50_ = 2.75 nM). The conjugate also displayed potent cytotoxicity for NCI-H520, NCI-H1581 and JIMT-1 cancer cells, although at higher concentrations (EC_50_ = 31.45 nM, 52.58 nM and 94.26 nM, respectively). Interestingly, COLO-699 cells, characterized by relatively high expression of FGFR1, were largely insensitive to T-Fc-vcMMAE (EC_50_ = 865.85 nM) (Fig. 4g and j). For all studied cell lines the conjugate was less toxic than the free MMAE, which is expected due to the well-known ability of MMAE to freely pass through cellular membranes (Fig. 4b–i). At the same time free MMAE displayed no selectivity, as it efficiently extinguished all analyzed cell types, independently of the presence or absence of FGFR1 (Fig. 4b–i). The specificity of MMAE is only observed when drug is linked to the T-Fc targeting molecule (Fig. 4b–i).

Since there was partially no correlation between FGFR1 expression and T-Fc-vcMMAE cytotoxicity, we analyzed with fluorescence microscopy the internalization of T-Fc into studied cancer cell lines. As shown in Fig. 4k no internalization of T-Fc into COLO-699 cells was detected, similarly to FGFR1-negative HCC-15 and T47D cells. The FGFR1-positive, T-Fc-vcMMAE sensitive NCI-H520 and NCI-H1581 cells displayed efficient uptake of T-Fc. These data suggest that T-Fc-vcMMAE is an effective and selective conjugate for targeting FGFR1-positive cancer cells. Furthermore, our data implicate that, besides target expression level, also dynamics of target cellular trafficking should be considered during selection of the molecular target and cancer types for development of efficient ADCs.

## Discussion

ADCs represent an important and rapidly increasing class of drugs. Up to date, FDA (U.S. Food and Drug Administration) approved ten antibody–drug conjugates directed against distinct molecular targets in different tumors (Zhao et al. 2020; Nobre et al. 2019; Syed 2020; Chang et al. 2020; Keam 2020; Markham 2020), and a large number of ADCs are under clinical development. Although FGFR1 represents an attractive molecular target for development of specific ADC, so far there is no ADC approved for treatment of FGFR1-overproducing cancers. Up to date, our group has reported several promising anti-FGFR1 cytotoxic conjugates, consisting of antibody fragments or FGFs as targeting agents, however their therapeutic potential requires further assessment (Sokolowska-Wedzina et al. 2017; Krzyscik et al. 2017; Lobocki et al. 2017a; Świderska et al. 2018).

We have recently engineered tetravalent anti-FGFR1 antibody, T-Fc, that displays several features highly desirable for the targeting molecule. First, T-Fc is highly specific towards FGFR1 and binds the receptor with very high, subnanomolar affinity. Second, the T-Fc displays very efficient internalization via receptor-mediated endocytosis and rapidly traffics to lysosomes where both T-Fc and FGFR1 are degraded. Third, T-Fc-mediated FGFR1 clustering on the cell surface simultaneously activates several distinct endocytic pathways, both CME and CIE. It is of high importance due to the fact that cancer cells are able to preferentially shut down CME, a pathway employed by bivalent antibodies typically used in the ADC approach (Fielding and Royle 2013). The T-Fc may overcome this limitation of conventional antibodies and assure sustained drug delivery to the tumor cells. Fourth, the T-Fc has large size, excluding it from rapid renal clearance. Fifth, the presence of the Fc region within T-Fc, interacting with the neonatal Fc receptor, may extend its plasma half-life (Liu 2018). Finally, T-Fc can be efficiently overproduced and purified to homogeneity.

All these highly promising characteristics of T-Fc prompted us to evaluate its potential in the form of ADC. We have successfully conjugated T-Fc with the valine-cytruline bearing cytotoxic payload, monomethyl auristatin E, yielding T-Fc-vcMMAE and have confirmed that the properties of T-Fc towards FGFR1 are not affected by the site-specific incorporation of MMAE. Furthermore, T-Fc-vcMMAE displays high cytotoxicity to FGFR1-producing cells, including lung cancer cells with EC_50_ in the nanomolar range. At the same time T-Fc-vcMMAE is virtually not toxic to cells devoid of FGFR1, highlighting the selectivity of obtained conjugate. The obtained EC_50_ values for T-Fc-vcMMAE are one of the lowest for FGFR1 targeting conjugates reported in the literature, confirming effectivity of the conjugate (Sokolowska-Wedzina et al. 2017; Krzyscik et al. 2017, 2019, 2020; Świderska et al. 2018; Lobocki et al. 2017b). However, additional modifications of T-Fc-vcMMAE are possible, like PEGylation, site-specific incorporation of additional warheads, linker adjustments to further improve efficiency of the developed conjugate. Cytotoxic conjugates targeting other cell surface molecules display wide range of EC_50_ values that are either lower or higher than the ones obtained for T-Fc-vcMMAE, which suggests significance of the molecular target, attached drug, DAR and cancer cell specificity for efficiency of the treatment (Khongorzul et al. 2020; Chiang et al. 2020; Andreev et al. 2017; Oller-Salvia 2018). Still, further tests, including in vivo experiments are required to confirm high efficiency of the T-Fc-vcMMAE conjugate in combating cancer in the living organism.

Typically, highly internalizing proteins that are overexpressed by cancer cells are selected as molecular targets for conjugate (Tashima 2018). One of the critical steps for effective conjugate action is their internalization via molecular target-dependent endocytosis. There are several reports focusing on the correlation between target expression and the uptake of conjugates. In most cases higher target expression promoted internalization of target-specific conjugates (Sharma et al. 2020; Tang et al. 2019; Beck et al. 2017; Rudnick et al. 2011; Damelin et al. 2015). Up to date, there have been no reports attempting to link FGFR1 expression to the internalization of target-specific conjugates and the induced cytotoxic effect. In the present study we have shown that the efficiency of FGFR1-specific molecule internalization, and not just the level of FGFR1 expression, correlates with the cytotoxicity of the conjugate. Our data suggest that endocytosis of FGFR1 in distinct cell lines can be differentially regulated, leading to an immobile pool of the receptor trapped on the cell surface. For example, lung adenocarcinoma COLO-699 cells characterized by relatively high FGFR1 levels are largely unable to internalize T-Fc-vcMMAE and are therefore resistant to the treatment with T-Fc-vcMMAE. Based on these observations, we suggest that not only expression level of the target, but predominantly its cellular trafficking should be considered when selecting molecular targets and cancer types for treatment with cytotoxic conjugates.

## Conclusions

Our data confirm the applicability of T-Fc in the form of ADC as a highly effective drug delivery vehicle for targeting FGFR1-producing cancer cells. The T-Fc-vcMMAE conjugate presented in this manuscript efficiently kills cancer cells bearing FGFR1 protein on their surface, while it is neutral to the cells that lack this receptor. Furthermore, we postulate that multivalent targeting molecules, due to high affinity for receptors and superior cellular uptake, may constitute attractive alternatives to conventional bivalent antibodies as drug delivery vehicles in the ADC approach.

## Data Availability

The datasets used in this study are available from the corresponding author on reasonable request.

## Abbreviations

ADC: Antibody drug conjugate
BLI: Biolayer interferometry
CIE: Clathrin-independent endocytosis
CME: Clathrin-mediated endocytosis
DAR: Drug to antibody ratio
EC_50_: Median effective concentration
ERK1/2: Extracellular signal-regulated kinase 1⁄2
Fc: Fragment crystallizable region
FGF: Fibroblast growth factor
FGFR: Fibroblast growth factor receptor
IgG1: Immunoglobulin G1
RTK: Receptor tyrosine kinase
scFv: Single chain variable fragment
T-Fc: Tetravalent anti-FGFR1 antibody
TCEP: Tris(2-carboxyethyl) phosphine
vcMMAE: Maleimidocaproyl-Val-Cit-PABC-monomethyl auristatin E
